# Connections on the Line: Interactions Between Care, Expertise, and Anonymity in a Suicide Hotline in Hong Kong

**DOI:** 10.1007/s11013-025-09954-z

**Published:** 2025-10-16

**Authors:** Nicole Zhong, Zhiying Ma

**Affiliations:** 1 The College, The University of Chicago, 5801 S. Ellis Ave, Chicago, IL USA; 2 Crown Family School of Social Work, Policy, and Practice, University of Chicago, 969 E. 60th St., Chicago, IL USA

**Keywords:** Care, Suicide, Expertise, Anonymity, Strangers

## Abstract

Suicide hotlines as a form of teletherapy have been associated since conception with questions of non-consensual reporting and intervention. This article investigates how suicide hotline volunteers in Hong Kong engaged with expertise and anonymity to perform care. The analysis consists mostly of semi-structured interviews with volunteers at a multilingual suicide hotline in Hong Kong. Firstly, we deconstruct expertise in hotline care—volunteers separated enacting expert knowledge from authority to simultaneously respond to cultural nuances in caller expectations toward authority and perform non-interventive care. Secondly, we show how anonymity within hotlines creates obscurations between callers and volunteers that raises the stakes to volunteers’ decision-making in care. When volunteers embraced uncertainty and committed to providing confidentiality and control to callers, anonymity facilitated the prioritization of caller choice, even in death. Finally, we reflect on how acts of volunteers recognizing and forming connections with callers can demonstrate how we can meet each other, bridge divides, and connect as strangers in the city.

## Introduction

Despite only having met for 30 min through voices and words, it was unexpectedly easy for Nicole to talk deeply with Charlie. Charlie was open and genuine about his experiences as a volunteer operator in AmaryllisLine,^1^ a multilingual suicide hotline organization in Hong Kong, and the warmth and thoughtfulness in his cadence was reassuring. When conversation pivoted to Hong Kong’s mental health landscape, Charlie spoke on our inaction toward caring for strangers or peers. He said^2^:

Mental health, especially out in the public… tends to be more extreme in nature, and I think there’s people who often shy away from engaging… I think it’s more that people actually lack the courage to step out. And I think we as Asians, as in [we] live in an Asian society, tend to let people deal with these issues by themselves. And I wish that more… as in… the stranger portion, I guess all of us…it’s really this courage building I wish that the ramp-up time is less for everybody, I would say.

A few months past the 25th anniversary of Hong Kong’s handover from UK to China and after the large-scale anti-government protests and ensuing oppressions, this conversation with Charlie felt especially relevant toward the fear, instability, and continued anxiety of change permeating the city. Charlie’s hope for courage building among strangers is not unique. In most suicide hotline calls, volunteers and callers are strangers, yet are compelled to treat each other as friends or close confidants. How do connections form between strangers with voices and words, in a society marked by ethnic diversities, wealth disparities, political tensions, and emotional distress?

## Situating Caregiving in Suicide Hotlines: Expertise and Anonymity

Suicide hotlines are typically single or occasional-use telephone lines that anyone can call to receive emotional support by a rotating group of volunteer or staff call operators; they are services that simultaneously distances and encourages deep connection. The comparative accessibility and immediacy of phone calls makes hotlines internationally recognized as an integral aspect of suicide prevention aimed at crisis response (WHO, 2014).

Since hotlines are a widely recognized form of suicide response, callers often considered hotline volunteers as experts, insofar that they expected volunteers to advise them in calls. However, while volunteers are experts insofar their use of intuition (Luhrmann, 2001), accumulated experience (Dall’Alba, 2018), and training and certification (Carr, 2010), they renounced their expertise to remain non-authoritative and prioritize caller choice. Anthropologists have explored tensions between the qualities of care enacted based on intuition versus standardization (Krishnamurti, 2021; Luhrmann, 2001), between how expert knowledge interacts with choice and intervention (Mol, 2008), and how experts have co-opted care-language to expert authority (Ma, 2020). Rather than seeing these aspects in tension as mutually exclusive, we view them as malleable and reflexive performance.

The practice of mental health care is heavily entangled with expertise, through institutional processes like diagnosis or licensing. These processes also perpetuate the authority of experts as the creators and holders of ‘irrefutable truths’ (Carr, 2010). On one hand, these ‘irrefutable truths’ trickle down toward creating public preconceptions–often pathologization–toward people with mental illnesses (Lester, 2019). On the other hand, ‘irrefutable truths’ are alterable: they are specific to cultures and groups (Kleinman, 1987) and can be redefined by experts and lay alike (Eyal, 2013). In our analysis, these truths, or what we refer to as *expert knowledge,* are both knowledge volunteers gained from training and experience. In contrast, *expert authority* refers to the perceived aura constructed through both volunteers’ own status as experts and associations to professional mental health experts. We trace how volunteers’ expert knowledge is reflexively shaped by institutional and intuitive knowledge; how volunteers use intuition to subtly enact or renounce their own expertise in response to caller perceptions on authority; and how such selective performance enables care.

In addition to expertise, we show how volunteers navigate care and connection within anonymity Anthropologist Lisa Stevenson argued that, under pressures to contribute to suicide prevention in immediate and dire situations, *care* is rendered anonymous so that it is not hinged on bonds between specific individuals like traditional therapies. This type of care within hotlines, which she calls *anonymous care,* discards people’s individual importance through standardization under the project of preventing death (Stevenson, 2014). Scholars have pointed out the hotline’s ability to either create closeness in its open-ended nature or alienation through its lack of presence (Zeavin, 2021). Under different care needs, the nature of a hotline’s anonymous intimacy has shown to be either ill-fitting–when the need for healing requires consistency, time, and recognition (Backe, 2018)–or uniquely suitable–when removing barriers to care by anonymizing volunteers’ identities and professions (Karlin & Hodge, 2022) or when protecting the identity of callers and volunteers to process shared experience (Mckinney, 2020).

The ephemeral and obscuring effects of anonymity on the line reflect stranger sociality in urban landscapes. Toward strangers, who are characterized through their un-belonging (Simmel, 1908/1971), care can be withheld as self-protection (Lee, 2014; Wu, 2021a). In the face of rising stranger societies, scholars have investigated how anonymity on the internet encourages extended relationships and intimacy without needing physical or nonverbal cues (Bargh & Mckenna, 2004; Aelbrecht, 2022). As another anonymous and ‘untraceable’ but open space, hotlines simultaneously simulate unrecognizable-ness inherent to strangers and allow strangers to freely meet in un-ordinary circumstances. The connections AmaryllisLine volunteers made with callers demonstrate how a (fleeting) intimacy between strangers is possible.

In this article, we explore how volunteers strategically engage with expertise and anonymity in order to perform listening-centered care within the hotline space. Responding to the nuances of callers, conversations, and expectations toward authority, volunteers made decisions in-the-moment of calls to enact expertise through guidance from their experience-built intuition. Within anonymity, volunteers radically accepted uncertainty to connect with callers, past boundaries, together, and in the dark. These strategies for building connection help us understand how care is practiced with expertise and anonymity, especially in the context of an increasingly prevalent stranger society.

## Methods

The analysis comes from interviews, each averaging an hour and a half, with ten active AmaryllisLine volunteers during the summer and fall of 2022. Participants had different backgrounds and identified as Cantonese, Singaporean, Chinese, French and British. Volunteers’ ages ranged from mid-twenties to mid-sixties and volunteering time varied from one to over twenty years. During interviews, Nicole asked volunteers about their experiences at AmaryllisLine and outlook on mental health and suicide in Hong Kong. While Nicole visited the hotline center and conducted a few in-person interviews, most interviews were on Zoom. In interviews where participants requested cameras off, the Zoom call imitated a phone call, potentially influencing how volunteers approached conversation (Briggs, 1986). Besides interviews, organizational websites and reports, and training presentations were reviewed.

Without understating research toward effectiveness and rectifying less satisfactory hotline practices and caller experiences (Chan et al., 2018; Mishara et al., 2007; O’Riordan et al., 2022), we focus on volunteers’ caregiving practices and experiences to explore alternative models toward understanding effectiveness in mental health. Like how hotline volunteers learn to not dwell on the success of their calls, we leave effectiveness open, flexible, and up to interpretation.

## AmaryllisLine and Hong Kong’s Mental Health Landscape

The history of mental healthcare in Hong Kong has been entangled with changing forms of authority and social belonging. During the start of British colonial rule, individuals with mental illness in Hong Kong were legislated as ‘lunatics,’ and their management was monopolized by authorities. Following practices of repatriating convicts, mentally ill patients were temporarily detained in asylums before being forcefully repatriated from Hong Kong based on ethnicity to London or Canton, China. Such practices blurred the lines between mental illness and crime, segregating those with mental illness and rendering them as social outcasts. As the city prioritized maintaining port business and ‘orderly and civil’ appearances, these strategies for managing mental illness set the basis for mental health care development as entangled with authority and determined through one’s compliance, participation, and efficiency within society (Wu, 2021a, 2021b, 2022).

In the 1960s, Hong Kong’s rapid urbanization led it to have the highest urban population density in the world (Lo, 2003). Under this landscape of stranger society, people who had mental illness, were neurodivergent, or experienced suicide ideation (SI) were feared for danger and dismissed for irrationality. In 1982, the Un Chau Estate Tragedy, where a person discharged from a psychiatric hospital stabbed several people, aggravated stigma toward mental illness.^3^ While the event spurred developments in community mental health services, these developments were motivated by distrust, preventing violence, and tightening control over patients (Chiu et al, 2000; Lo, 2017; Yip, 1998, Wu, 2022).

Given these historical backgrounds, psychiatry in Hong Kong has relied on building professional authority and institutionalizing patients. Despite attempts to follow the UK in their successful deinstitutionalization movement, increased public stigma against mental health halted the development of community-based services out of fear (Wu, 2022; Yip, 1998). Indeed, the transition from hospital-based, institutionalization of mental health to more community-based services has only been a recent development for the city within the past two decades (Chan et al., 2015).

From 156 years as a British colony and brief Japanese occupation to being a special administrative region of the People’s Republic of China, Hong Kong has a long history of losing autonomy and authority. In 1997, Hong Kong’s handover from Britain to China led to both heavy out-migration, illegal immigration (Manion, 2004; Murphy, 1991), and political turbulence, which increased fear and alienation within the city. Following the 1998 Tiananmen Massacre, divisions grew between the Pro-Beijing and the Pro-democracy camps (Lui & Chiu, 2000). In the 2000s and 2010s, political instability with policies imposed by Beijing resulted in further protests, escalating with the Umbrella Revolution in 2014, the 2016 Independence Protest, and most substantially, the 2019–2020 Hong Kong Protests (Gunia, 2019). These protests caused several splits—Mandarin speakers and people from mainland China were seen with suspicion, and openly supporting either political camp could lead to ostracization from the other side (Loong-Yu, 2020). Particularly, the 2019–2020 Hong Kong Protests and the following implementation of the National Security Law have created an environment of political uncertainty and instability. Notwithstanding PTSD from protesters, this political tension and instability, including the conflicts with peers, fear for safety, and political hopelessness has led to a public crisis of mental health (CECC, 2022; Lau et al., 2017; Li, 2021).

These political shifts and turmoils have reasonably affected locals’ regard for anonymity and the hotline. While hotline volunteers observed people calling to express fears or experiences of ostracization they also noticed that despite the line’s anonymous nature, callers, fearing the line being tapped, were increasingly reluctant to share political opinions. Anonymity also expanded the hotline’s caller base: in particular, callers from Mainland China who used the line because of how its primary use of English strengthened its presumed distance from government surveillance.

Given the city’s political and mental health histories, people in Hong Kong have developed a complicated and uneasy relationship with authority. On one hand, the force of authority against people’s agency regarding their health and wellbeing is feared (Li et al., 2021; Wu, 2022). On the other hand, parts of authority practiced in institutional mental health are expected and welcome for its more directive guidance and alignment with Confucian values (Yip, 2000). As a hotline that practiced Western-originated care models, AmaryllisLine volunteers encountered tensions around differing cultural expectations for authority.

In addition to such stress and tension, contemporary understandings of suicide make it complicated to seek care. Culturally, while family members tend to rely on each other for care, Chinese cultural attitudes in Hong Kong were shown to regard suicide as an irresponsible or immoral act of filial abandonment and breaking social cohesion (Chow & Yip, 2011; Fei, 2011; Lee et al., 2007a, 2007b).

Government funding and efforts were made toward public mental health services and education—from the Hospital Authority, Social Welfare Department, or Health Bureau Hotline—in Hong Kong (Hong Kong Free Press, 2023; Collins, et al., 2006; Legislative Council Commission, 2024), but efforts were still overwhelmed by city’s immense mental health need (MindHK, n.d.), leading to scarce, overloaded, inaccessible, and hastily given care (Chan et al., 2015). Hong Kong social workers, historically contributing to political activism, were also increasingly bureaucratized, especially following policy implementations effective following the 2019–2020 protests that led to demoralization and burnout (Leung, 2024, 2025; Chui & Gray, 2004). As a (non-governmental) part of the city’s mental health efforts, hotline volunteers were quick to comment on these barriers. Kristina, a 5–6-year volunteer in her late 60s, recalled:

And I think the government system…they’re so overloaded…And I think those who even think personally about how the things should be done have to apply by the system and the structure…and people feel that, they feel like they’re just on a conveyor belt, they’re not getting enough time, and they’re not getting individual care.

Moreover, in the mainstream mental health system, people are unable to get specialized attention and treatment for their differing needs unless they threaten to disrupt the public order. Consequently, the individual needs of people are generalized and standardized, creating a landscape akin to anonymous care (Stevenson, 2014). Elena, a volunteer leader, shared similar sentiments to Kristina, and elaborated on the specific experiences of callers:

[...] A doctor…who is one of the volunteers, he said, ‘You got to rock up with a knife almost for them to…help you,’…So we get a lot of calls like that. Say, ‘You know, my doctor doesn’t really listen. My doctor has no time,’ and they’ve got no money to go anywhere else[…]

The shortage of available doctors and time meant that individuals had to endure long wait times and appointment time restrictions, which made it impossible for people to feel fully seen and cared for. Within these continuing practices of generalized and standardized care, individualized connection and attention was both desired and increasingly hard to come by.

## An Introduction to AmaryllisLine: A Listening Approach to Care

The AmaryllisLine center was located within a small but cozy apartment complex where staff and volunteers could eat, take breaks, and gather. A small administrative office, a large kitchen with free food, and a hallway to individual call rooms connected from the main living and dining area. Lastly, the center had an east-facing balcony extended to an unobstructed, calming, and breathtaking view of Hong Kong’s mountains and sea. While volunteers had begun to take calls from home, most volunteers still had most of their shifts at the AmaryllisLine center. For some volunteers, the center offered both stabilizing physicality in contrast to the nebula of the phone and a place for volunteers to ‘leave their emotions’ after shifts. The physical backdrop of the center, the organization’s structures, and volunteers’ communication styles all took part informing each call.

Volunteers in AmaryllisLine typically had one 3–4 h day shift each week and one 5 h night shift each month, where they had on average 3–4 calls. Typically, a shift had either one or two volunteers operating, with overnight shifts operated by a single volunteer. Volunteers’ commitment to these shifts allowed AmaryllisLine to be open 24/7, 365 days a year.^4^ In addition to calling, the hotline also offered an email service. Volunteers were wide-ranging in backgrounds and motivations. Many volunteers had jobs unrelated to mental health, but others were training or practicing mental health professionals.^5^ AmaryllisLine volunteers were restricted from disclosing their volunteer status outside of direct family by organizational policy to keep volunteer identities anonymous.

The training to become expert-certified as a volunteer was rigorous and strict. Over a few months, volunteers-in-training (VTs) met weekly for interviews, tests, lessons, and conversation practices. In training, VTs learned conversational skills–listening, non-advice, and communicating warmly–and knowledge on mental health, suicide, and their context in Hong Kong. They practiced commonly encountered caller situations, including financial difficulties, sexual assault, and abuse. VTs were required to strictly follow ethical principles specific to AmaryllisLine, particularly in upholding caller choice and their ability to choose death.^6^ After passing training, VTs entered a probation period where they answered real calls with an experienced volunteer. Only after final approval from the volunteer could VTs take calls on their own.

While AmaryllisLine primarily supported people struggling with mental health, suicidality, or in crisis, they received callers for other support. These included callers practicing English, sex callers, prank callers, venting callers, repeat callers, callers looking for casual conversation, and more. Volunteers had categories for describing these different types of callers and expectations and methods of approach toward them.

AmaryllisLine self-marketed as a multilingual hotline, meaning that they received callers that most often spoke English, Mandarin, Cantonese, and, sometimes, other languages. Volunteers attributed the language variation among callers to both Hong Kong’s multilingual diversity and callers abroad seeking operators who understood Asian culture. Although most volunteers could speak Cantonese, English was both AmaryllisLine’s only language requirement and the language used in training. When volunteers’ and callers’ languages did not match, volunteers would tell callers to redial during a shift-time with a corresponding multilingual speaker and re-open the line for a (hopefully) lingually matching caller.

The methods and ethical principles of AmaryllisLine were strictly non-interventive. Volunteers used a twofold approach to support, i.e., listening and non-advice giving, which framed their care within hearing and understanding the caller’s positionality, choices, and wants. In its most extreme form, this method of non-interventive support meant that if a caller was determined to die by suicide, AmaryllisLine volunteers respected callers’ choices of how they wanted to live and end their lives. AmaryllisLine volunteers committed to never calling authorities on callers and would only consider the possibility if callers themselves asked for it.

Listening was the basis of how volunteers gave and conceptualized care. Kristina described active listening to Nicole: “...we listen to not only what they’re saying but also how they’re saying it, we assess the whole emotional state of the person...”. As each caller was a new person, listening was crucial for volunteers to individualize support and create genuine connections. When asked how she would approach callers who were ‘on the verge of suicide,’ Kristina immediately responded that “the best way is to listen to them.” Listening was both an opportunity for callers to express themselves and for volunteers to understand callers: a conversational method for conveying care within crisis.

Listening required unconditional openness to callers’ words. “They’re free to talk about anything they like, you know, whether it makes sense or not,” Kristina answered when asked about speaking with callers about politically sensitive topics, “We’re there to listen, and we don’t have an opinion on anything...we’re like a sounding board.” By reflecting back callers’ language and positionality, volunteers centered callers’ perspectives through listening.

As much as listening was unconditional, volunteers balanced their personal investments with listening to sustain care. Elena described:

[...] But yeah that’s what I mean, this detachment. It’s not that I don’t care, I do care, and in order to really care and give you the support that you need, I mustn’t get too emotionally involved, I must listen, I must accept and have them really feel that I care [...]

In other words, listening could be unconditional and supportive because of the distance that volunteers took from their work—by setting aside the volunteer’s personal stakes and biases, volunteers could guarantee care. In another instance, Florrie, a volunteer pursuing a postgraduate degree in psychology, described feeling torn when her education informed judgments contradicting callers’ perspectives. Florrie described better accepting her judgemental feelings when she focused on callers’ perspectives over her own. Similarly, Kathie, a 13-year-long volunteer, described ‘emptying herself’ to be genuinely curious about callers’ viewpoints rather than simply acknowledging differences or feigning agreement to support callers.

Listening informed volunteers’ speaking roles in calls. Volunteers communicated through non-advice giving, leading callers to their own answers by asking questions, giving validations, and showing engagement. Non-advice giving ideologically prioritized caller autonomy, meaning volunteers could not interfere with callers’ decisions and would refuse even if callers asked for advice. Through listening and non-advice, volunteers cared for callers through centering, hearing, and understanding their thoughts and experiences.

## Deconstructing Expertise

When callers asked for advice in calls, volunteers explained that they were unable to give advice, as they were non-interventive and could not assume to know about callers’ lives. While many callers understood, others would get frustrated, highlighting the tension between authority’s potential for guidance versus force. As ‘experts’ that did not wish to enact expert authority (Carr, 2010), volunteers used intuition as a more subtle and flexible enactment of expert knowledge to reconcile caller expectations.

Through training, practice, and eventually over time, a wide exposure to callers–the unsung mental health experts–volunteers developed expert knowledge unique to their roles as hotline operators. As volunteers also accumulated often decades-long of experience, they became more adept at giving support. Hester, a 2-year volunteer, described, “I think it does make you a better listener…from experience—after a while, you’ll be like, ‘No, there’s something deeper, and I need to like–’...because you know there’s something that they’re actually not really telling you to your face.” We describe this experientially developed practice of ‘listening beyond the surface’ as intuition (Luhrmann, 2001).

Like Hester, many other longer-time volunteers attributed their preparedness and discernment toward callers toward experientially developed feeling and know-how. In Luhrmann’s (2001) study of psychiatric training, intuition denotes a psychiatrist’s ability to ‘feel’ a patient’s diagnosis and is perceived as a marker of expertise. As compared to use toward determining an ‘irrefutable truth’, such as diagnostics or treatment, volunteers’ aimed their intuition toward to carry out listening and non-advice, flexibly ‘feeling’ and responding to callers’ undercurrents in conversations (Carciun, 2018). Intuition attuned volunteers to what information, questions, or validations were most insightful or helpful to callers in-the-moment (Dall’Alba, 2018).

Volunteers used intuition to help subtly guide callers without threatening autonomy. When Charlie was asked about his limitations as a volunteer, he responded:

[...] I can prompt…and I can ask questions about how they feel about certain, uh, certain aspects of their situation…I will go through and hear their thought about it…and sometimes it sparks off a lightbulb by themselves…so…not outright giving advice but at least steer the conversations to some emotions that they may not have considered.

While training unified volunteers strictly, volunteers’ intuition flexibly addressed constant in-the-moment uncertainties and ambiguities introduced by callers. Intuition helped volunteers feel out which non-advice prompts could lead callers toward new considerations. Elena mentioned that when a caller said they wanted to end their life, she would ask, “I wonder whether it’s not so much a case of wanting to end your life but rather that you want a different life?” Elena found that callers would agree with her statement more than their own. Elena’s usage of non-advice unearthed what she intuited from callers: the hidden desire for support for underneath the surface of what they were saying.

Volunteers also felt ambiguities toward the extent AmaryllisLine policies could apply to specificities of callers’ situations. As a result, some volunteers opted to bend organizational rules to support callers in ways they intuited would be more suitable. One instance of rule-bending was AmaryllisLine’s policy to ask every caller if they experienced SI. While Silvia, a 10-year volunteer, always asked, using intuition to creatively transition to the question, Jessy, a 2-3-year volunteer, mentioned the opposite: “Mmmm, if the call is long enough, I will do it. If I know that the person is just venting during their work break…I don’t want to slow them down by asking...” Jessy saw on call logs that others also did not inquire about suicidality in many calls. While Silvia’s decision upheld AmaryllisLine’s principles and offered nervous callers chances to share SI, Jessy believed her decision demonstrated consideration toward call time. Volunteers interfaced with intuition to manage ambiguities, accept uncertainty, and be flexible about organizational ‘truths.’

The mode of intuition as less ‘truth-centered’ relates to volunteers’ commitments to non-intervention and rejecting expert authority. AmaryllisLine volunteers were all quick to deny their expertise, unlike more certified mental health professionals. Jessy described, “We don’t have professional experience right, and we should never forget that what we know about a person is what they tell us.” Interventions from professional mental health care can be supportive but also “[reinforce] the presence of experts, knowers, and authorities…” (Rosenbaum & Liebert, 2015, p. 190). In contrast, as Jessy described, volunteers recognized callers as the biggest experts of their lives, creating a necessity for callers to participate and authorize the caregiving process.

Volunteers who were also mental health professionals intentionally separated their professional and volunteer expertise. Kathie, a professional counselor and volunteer, shared:

[...] we are trying to answer their need and try to help them to express their frustrations or make a companion with them, so it’s different [from counseling] because for hotlines we won’t have the expectation that we help them solve their problem or find solutions for them.

In contrast to professional mental health in Hong Kong, which centers on expert authority (Yip, 2004), hotline support for Kathie meant detaching from counseling work to be a companion first. Kathie’s rejection of expert authority in a lack of ‘expectation for solutions’ allowed her expertise to be flexible toward callers’ needs.

Some callers expected direct expert guidance from volunteers, calling specifically for advice or who would get angry or insistent when volunteers refused to give them advice. It was unclear whether these expectations were Hong Kong specific, but, aligned with scholarship on transcultural care (Crozier, 2018; Zhang, 2014), a few volunteers related caller expectations to general cultural backgrounds. Florence, a volunteer in her sixties who joined after the SARS epidemic, described these cultural differences:

[...] Native English speakers, Westerners, expats, they like this kind of approach that, you know, you don’t tell me what to do, because ‘when I talk to friends, they do that, and that isn’t something that I’m looking for.’ …but when we talk to Chinese callers, some get angry or upset that we don’t tell them something…And very often they say…I can just talk to a tree hole…or a wall if you’re just listening...So they really would like you to give them a direction. Something to share. I think that is generally a very obvious difference.

Under Florence’s observations, assumed Western callers expected non-advice as a ‘professional’ and subtler communication of expert knowledge. Meanwhile, assumed Chinese and Cantonese callers expected volunteers to act as expert authorities or give advice as more knowledgeable peers.

Volunteers’ adherence toward non-advice despite caller requests otherwise could be seen as unsolicited Western cultural authority. Florence forwent AmaryllisLine rules by using her intuition to resolve these conflicts:

We may chat more like friends^7^…Officially, we shouldn’t, as this is not a chat-line…when I handle, you know, local callers who are really expecting something from the volunteer, I may give them encouragement; now, even to give encouragement is not something the AmaryllisLine wants us to do. Somehow I have to…give them a bit of light […]

For Florence foregrounding casual intimacy offered an alternative way to provide callers with direction without resorting to ‘irrefutable truths’. By self-positioning as a ‘friend’ and speaking in a chatty style, direct or encouraging remarks may be contextualized with more relational equality and less authoritative weight. Here, treating callers as friends provided an alternative way of approaching advice giving—through the viewpoint of relational intimacy rather than a hierarchical one of an expert authority.

While volunteers disassociated with expertise, callers assumed that volunteers had some advice or expert knowledge to offer. Intuition, drawing from different truths and experiences, allowed volunteers to enact expert knowledge while ‘de-legitimizing’ truth by bending organizational rules and adapting to callers’ personal needs. By doing so, volunteers could step out of authority toward a more relationally intimate mode of care.

## Connections amidst Uncertainty and Anonymity

The lack of authority volunteers held toward callers was also in part due to how anonymous limited volunteers to singular chances to understand and interact with callers, raising the stakes of many factors of conversation. As a result, decisions volunteers made toward how to respond to callers was simultaneously significant and uncertain.

**Fig. 1:**
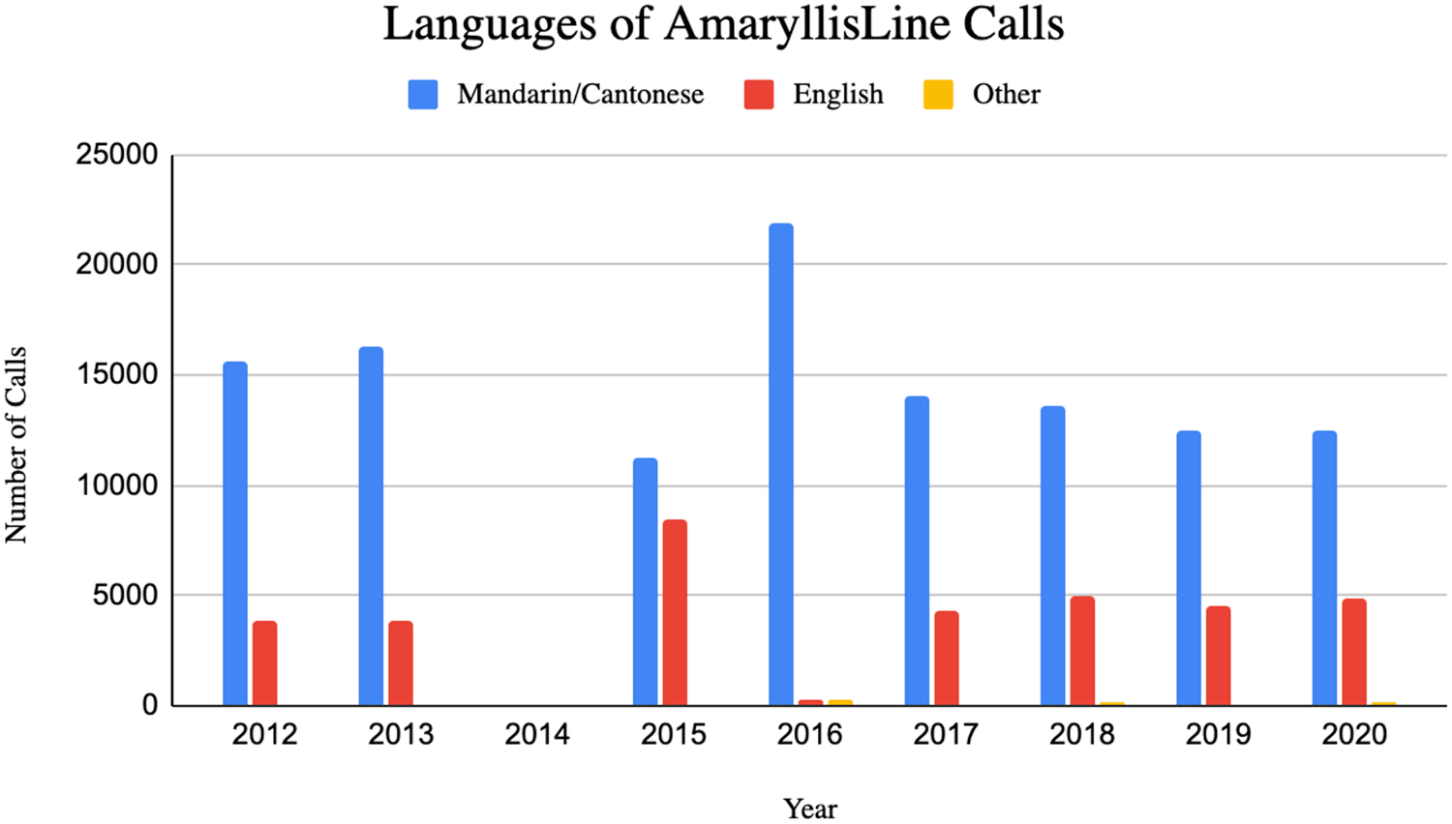
Language distribution of AmaryllisLine calls in recent years. There are more Mandarin/Cantonese language calls compared to English each year. *No available data for 2014. Alt Text: A bar graph titled ‘Languages of AmaryllisLine Calls’ showing a distribution of the number of Mandarin/Cantonese, English, and other language calls yearly from 2012 to 2020. Image Description: Shown is a horizontal bar graph titled ‘Languages of AmaryllisLine Calls’ showing a distribution of the number of Mandarin/Cantonese, English, and other language calls yearly from 2012 to 2020. The range of the y axis, titled ‘Number of Calls’, in the graph is from 0 to 25,000 calls. The rounded number to the thousands of Mandarin/Cantonese calls are around 15,000 for 2012, 16,000 for 2013, 11,000 for 2015, 22,000 for 2016, 14,000 for 2017 and 2018, 13,000 for 2019 and 12,000 for 2020. For English calls, the rounded numbers for calls are around 4000 for 2012 and 2013, 9000 for 2015, less than 1000 for 2016, 4000 for 2017, and 5000 for 2018, 2019, and 2020. The rounded number for calls in other languages are less than 1000 and barely visible in the chart for all the years. There is no available data for 2014. Figure Data Info: The data for the figure were compiled from organizational annual reports. The exact methods of how calls were counted is unknown.

Of the many heightened stakes volunteers dealt with in conversation-making, language was significant. In the hotline’s absence of physical presence or cues (Lester, 2021), callers and volunteers relied on language to connect past anonymity’s obscurations. However, in a multicultural city like Hong Kong which hosts a wide span of languages, AmaryllisLine’s volunteer shortage meant they were unable to cover English, Cantonese, and Mandarin every shift. Therefore, while volunteers sometimes coincidentally knew callers’ preferred languages, they otherwise compromised communicating with less fluent languages. As callers and volunteers typically spoke about hard topics, unfamiliar languages without nonverbal cues created caregiving challenges (Fig. 1).

Such language mismatches were particularly difficult for English-only speaking volunteers, where 5 h shifts sometimes consisted fully of asking callers to redial. AmaryllisLine’s inaction toward adjusting language requirements or English-only shifts despite self-marketing as a multilingual service meant that it generalized callers under a single cultural and linguistic umbrella. As they were turned away from the line, Non-English callers were heard but not understood; anonymity worked against them to prevent any sort of recognition.

‘Venting’ callers, or callers who vented without expecting any reciprocal conversation, were exceptions language-wise for English-only volunteers. English-only volunteers could intuit these callers’ intentions to vent and listen to callers’ emotions without understanding them, creating an avenue for care through anonymous preconceptions of volunteer-caller roles, listening, and intuition.

As shown by these language mismatches, anonymity prevented volunteers from predicting any aspect of callers. Hester reflected:

[...] even now, I still get a bit scared when the phone rings, because you have no idea who’s gonna pick up…you could have like a very quiet day and suddenly—Bam! You get a really suicidal caller…every caller is different…there’s always that little element of unpredictability that keeps us on our toes.

The simultaneous uncertainty and individual effects callers had on a shift’s trajectory compelling volunteers alertness toward callers’ individual characteristics. Eventually, experience with uncertainty led volunteers to be less assumptive. When Kathie was asked if volunteering changed her views, she recalled a caller with a perspective unimaginable to her, saying, “...so, I guess after this many years, I learn more about not to make assumptions…Don’t think people should do this or should do that because they are very different...” Experience helped volunteers catch personal and medically standardized preconceptions. As volunteers ‘made assumptions’ about callers with intuition to guide conversation, they also practiced non-assumption–to reject authority–to give care under anonymity.

Kathie’s non-assumptive stance aligned with AmaryllisLine’s goal to support callers over preventing death. Charlie reflected:

[...] I think it’s both empowering and it’s also—as in if you are outside of this [call space]—it’s very frowned upon...And we will try our best to help, we try to have resources, and at the same time, if the person rejects us from all the help that we want, we will not actively intervene because it’s their—they choose how they live, or die…it’s always pain—[sic] it’s always difficult because naturally as humans we don’t—we want to save people from dying, it’s counterintuitive […]

All interviewees were against non-consensual reporting regardless of caller situation. While volunteers felt the difficulty of prioritizing callers’ needs over their instinct to save lives, they understood the importance of recognizing what callers own choices. Counter to how *anonymous care* universally prioritized staying alive (Stevenson, 2014), volunteers’ commitment to non-assumption prioritized callers’ choices, even for death.

The assurance that their information will not lead to physically altering consequences contrasted the control and authority practiced in traditional psychiatry within Hong Kong (Yip, 2000, 2004) and could offer individuals uncommon breathing and talking room–to be listened to, sometimes for the first time. Florrie described what she might communicate to a caller determined to die by suicide:

[...] don’t say ‘uh you know what suicide is wrong,’ but show the person that if you have indeed decided it, we also respect that, [and] we’re here for you because you called us for a reason, and if you think calling us might help you for anything, then we’re just here to listen to you, and that’s it [...]

Listening and understanding callers’ choices was how volunteers cared for callers in these dark moments. Offering a caller respect for such hard decisions helped volunteers recognize and connect to callers holistically beyond their SI and expanded AmaryllisLine’s scope from suicide prevention to all suicide-related conversations, cutting assumptions of suicide as a bad act.

The high stakes of language at the beginning of calls reflected the high stakes of choosing death as realities of hotline care heightened by anonymity’s original universal design (Stevenson, 2014; Zeavin, 2021). Volunteers’ radical acceptance of uncertainty, which characterized concessions with callers based on language—unthinkable under a hotline’s promise for universal care—also characterized accepting the choice for death.

### Strategies for Connection in a Stranger Society

AmaryllisLine volunteers had a far reach into Hong Kong’s communities—particularly those in the margins and grassroots. Contrasting with Hong Kong’s highly stratified and asocial environment, volunteers’ practice of listening in anonymity aimed to create connections to bridge distances between strangers in the city.

While AmaryllisLine reached callers from all different quarters of the city, by obscuring people’s identities, anonymity generalized caller experiences. Similarly to many volunteers, Florence described finding some of ‘far-reaching experiences’ to be “bizarre, amazing, surprising, and sad” and helped her feel grateful to “lead a pretty normal life”. While rewarding for volunteers to learn from callers’ life experiences, these self-comparisons could create distance, complacency, and generalize callers’ identities through a lens of hardship.

Yet, anonymity obscuring identities also allowed callers and volunteers to un-ordinarily meet.^8^ Upon further prodding into volunteers’ understanding of caller hardships as ‘reward,’ many reoriented reward as privilege to learn from and access Hong Kong’s diverse experiences. Florence reflected that if not for the hotline, she “would not be in contact with those people ever in [her] life.” Beyond physical and circumstances distances, Florence understood how her positionality made it difficult to hear from callers in different classes or marginalizations; she additionally expressed that she might feel prejudice if she saw a caller’s looks, attires, and manners as compared to just their voice. Obfuscation via anonymity opened Florence to understanding and empathizing with experiences she would otherwise ostracize.

Florence’s acknowledgment of her own prejudice was unsurprising considering Hong Kong’s highly stratified social reality. Beyond divides created from protests (Hong et al., 2025), rising levels of income inequality and job insecurity has created a socially polarized and distrusting environment (Hung, 2020; Lee et al., 2007a, 2007b) and the city’s diversity has been spatially and ethnically segregated (Lee et al., 2007a, 2007b; Lo, 2005; Siu-kai, 1997). These increasing divides have increased the boundary of distance that people need to cross to reach, communicate, or connect with each other.

The anonymity of the hotline made a space specifically for strangers to create extended interactions with each other. As with Florence’s experience, anonymity obscured identifiers that would have caused prejudice—flattening out social stratification and hierarchy within the call space. Extended interactions between urban strangers also generally requires high levels of cooperation (Karp et al., 1991). Volunteers appreciated callers’ leaps of faith: Florence said, “[It] always touches me to feel that they do want to talk to someone like me who they’ve never met.” Volunteers and callers were inclined to trust each other despite being strangers, creating an atypical social-boundary-crossing space.

While volunteers could access Hong Kong’s margins, their access was also inseparable from the structural circumstances causing the city’s social and class inequalities. Jessy recalled:

[...] I’m thinking of people who have like super hardcore debt, no job, and they know that no matter what when they hang up, the loan shark will come. And there is nothing because it’s so factual, and not so much in your mind and like, ‘Oh, you can look in a positive way.’ No, the guy is going to get his knees broken in probably one or two days, and there is nothing you can say that will make them feel better about it. So, those calls are super frustrating because you just feel like you wasted their time, and maybe they are even more frustrated than you.

Under the limited physical access or impact of an anonymous telecommunication service, volunteers had difficulty providing any tangible support toward callers’ overwhelming structural difficulties. These calls highlight pressing needs for more community mental health resources and, even more, systemic shifts. As volunteers could only access callers incorporeally and abstractly, they focused on being present together with callers through listening. Listening helped volunteers create connections to counteract the distances of the city’s inequalities and the line’s limitations.

Depending on the volunteer’s approach to listening, care could range from being objective and distanced to emotional and personal. When Nicole asked if Florence pitied callers, to follow up to her remarks about ‘being grateful through comparisons to callers’, Florence responded, “...I don’t have preconceived ideas about what I should or shouldn’t be doing. I do it the way we’ve been taught.” Trained to decenter their judgements, volunteers could detach personal feelings from their burden of care. Such objectivity may be appreciated for how it centers callers and prevents the personal qualities of volunteers from interrupting needed care. Yet, this assumed objectivity of listening prevented considerations for how inherent human biases may affect how volunteers intuit and choose to listen and offer non-advice.

When volunteers opened themselves to how their personal experiences and biases may affect conversation, they invited callers’ experiences into their own emotional lives. Jessy described:

Some calls…you can really relate to…the caller is like, ‘Yes, you understand me. You understand this frustration.’ And, well, I’m glad that helped, but then I have to face the fact that I have this unresolved issue…and the other side is that you have a caller that may not relate to you but they call you, and they are crying and screaming and you can feel how overwhelmed…they’re like ‘I don’t want to die, but I want to kill myself,’… there’s nothing you can do really except listening to them.

Rather than a limitation for its non-intrusive nature, listening was seen as what one *could* do, also because it did not require volunteers to produce any outcomes to create human connection. Listening allowed callers to share their painful, joyous, and messy realities with volunteers—creating human connection. Elena described, “...They call, but they don’t want to hear anything, so why are they calling?...They called for a human voice, they called for comfort in what might be their last few moments…” Elena observed the value of the emotional resonance and connection human voice gave to people during crises. She elaborated:

[…] it’s this human touch. Very, very, occasionally, if it’s a very tricky call, I say, ‘um, do you have a name you would like me to call you? It doesn’t have to be a real name.’ Sometimes, they say ‘No,’ I say, ‘well that’s absolutely fine,’ or they will give me a name: ‘That’s not my real name.’...But you’ve got something—like if you were in a terrible state and I said, ‘Nicole, it’s very heavy for you at the moment,’ and using that name, it makes you feel someone is recognizing you.

While anonymity obscured people’s public identities, aliases, as self-chosen representative identifiers, held the same weight and recognizability as ‘real life’ names. Although nested in different practices, Stevenson describes how, contrasting the hotline’s anonymous care, naming pulls at personal ties as care. Regardless of being dead or alive, calling a name was to be called in community with another person (Stevenson, 2014). In AmaryllisLine, naming in conversation gave care through recognizing the existing relation between volunteers and callers within anonymity’s socially flattened space.

Connections also helped callers understand themselves, when volunteers helped callers brainstorm, word out a solution, or name an experience. Silvia described what she sought out with callers:

I know that I’ve hit it in the right spot when they say, ‘That’s exactly right! That word,’...that’s when I feel like I’ve been able to hear what the other person is saying, and I’ve been able to reflect back to them what I think they were feeling and for them to say ‘Oh my god, that’s it.’

Creating verbal affirmations for callers required attentive listening to recognize the specificities of a callers’ experience. Volunteers believed that these connections facilitated needed relief from distress and gave callers strength to move forward. Kristina described:

For me it’s when their emotion changes. They’ve calmed down, and you feel like you’ve got some kind of rapport…and you can go on for as long as the caller wants to engage with you...If a call goes like that, it’s quite satisfying. It means that you were there when somebody needed you. And that’s the reason you signed up to do it […]

While positive outcomes were motivating to volunteers, Kristina’s observations highlighted her intentions to ‘be there for someone’—to provide company and human presence in of itself. Through close and attentive listening, volunteers could recognize and connect to callers regardless of being strangers with varied stratifications in the city.

Connections within anonymity also developed over time with AmaryllisLine’s repeat callers. As some callers regularly called the hotline, volunteers and callers increasingly recognized each other by voice and conversational topics. While these practices were not accounted for in AmaryllisLine’s original intentions or structures, the organization did not reject them (Backe, 2018; Mckinney, 2020) but rather coordinated care and created structures—including notes or tips from peers—to uphold the recognition of repeat callers for new volunteers. Still, repeat callers could be received by experienced volunteers who did not recognize them.

As repeat callers familiarized with the line, they used the service in a more personalized way; some called the same time every week, reminiscent of traditional talk therapy. Volunteers with consistent shifts could anticipate certain callers and vice versa.^9^ When AmaryllisLine volunteers recognized these callers, they could support more deeply without forcing familiarity, practicing distinct and close relationships in an indistinct and physically distanced space.

AmaryllisLine methodologies also faltered under regularity. As volunteer-caller relationships developed, giving unbiased support could become more difficult. Silvia spoke of her frustrations hearing the same issues for years from some repeat callers:

[…] it’s that they’re genuinely very vulnerable, and I want to be there for them, but they’re using us as their substitute friend. And I think that’s sort of the wrong message, on the one hand, but on the other hand, if we cut them off then they have nothing […]

Many volunteers described having ‘repeated conversations’ with callers. Under AmaryllisLine’s non-interventive methodology, if volunteers could not break callers out of subject matter, they ended up rehashing the same questions and answers. Anonymity’s obscurations both allowed these callers to repeatedly rehash their conversations and obstructed volunteers from knowing how to help callers beyond their scope of conversation.

Some repeat callers created deeper bonds with volunteers. Jessy described, “It’s more like keeping up with someone you like, and you have like more advanced conversations with them. And those ones are kind of a treat to have them on your shift when it’s someone you really enjoy, and it’s cool.” Although these relationships were not maintained beyond the hotline, their existence remained within and impacted volunteers and callers, demonstrating conjunction of ephemerality, anonymity, and lasting connection. It may be unintuitive to invest in ephemeral relations among strangers (Aelbrecht, 2022), yet repeat callers-volunteer relationships demonstrated willingness to build deep connections.

The relationships volunteers and callers built allowed volunteers to stray from AmaryllisLine’s methodologies by coordinating alternative care with callers themselves. “...After a while you kind of get a sense of what they like and what they don’t like…” Hester shared, mentioning repeat callers with chronic health conditions:

[...] sometimes, you know that certain days are better than other days, and bad days you kind of know from their tone and how they’re talking to you…So, for those callers, sometimes you end up talking about very random topics—you’re not supposed to…but I think with [repeat callers] because you kind of know that’s what they’re calling for, like they just want some distraction from life…I’ve had very interesting discussions…with some of our [repeat callers].

Through establishing individualized connections, volunteers could give more suitable support. Repeat callers and volunteers used the hotline as a tool to fulfill personal care needs, rather than let hotline policy dictate care. Anonymity and confidentiality facilitated this process—while risking misuse, volunteers could give support they were ‘not supposed to’ without consequence.^10^ Within Hong Kong’s capitalist and competitive environment, it is difficult for locals to form networks outside of their family, which has been particularly tough for the elderly, who have faced familial isolation from the pandemic and post-protest migration waves (Lau, 1981; Lee et al., 2005; Tao, 2024). The practice of giving such consistent and needed support showed how, despite the obscurations of anonymity, volunteers could become a part of callers’ support networks.

Within Hong Kong’s overwhelmed mental health landscape, volunteers embraced uncertainty toward outcomes to connect with callers fully over their ‘suicide risk’. As AmaryllisLine received callers from all different quarters of the city, anonymity both obscured identities and enabled boundary-crossing connections in the city’s stratified, polarized, and asocial environment. Within the inability and hopelessness of Hong Kong’s public and mental health crisis, volunteers used listening (Biehl et al., 2020) to recognize and be present with callers in the dark when physical support was impossible. These connections between callers and volunteers within anonymity demonstrated the possibility of personalized care in ephemeral but deep relational connections.

## Conclusion

In this article, we examined the approaches volunteers took toward providing listening care and connecting with callers in a suicide hotline. Within the frayed political and mental health landscape of Hong Kong, volunteers’ listening-centered care made efforts to answer the needs for connection in mental healthcare and among strangers in the city. In these efforts, volunteers constantly made decisions navigating expertise and anonymity to build trust and connections on the line.

Volunteers supported callers primarily through listening and non-advice giving, and used intuition to create subtle reframings and guide callers in nuanced and uncertain moments in the line. In accordance to AmaryllisLine’s non-interventive approach, volunteers positioned themselves as non-experts despite their certified and experiential expertise and intuition. Volunteers’ rejection of expertise, however, was complicated by callers seeking expert advice. The adaptations volunteers made to approach callers as friends suited caller needs for direct engagement without imposing expert authority and created intimate connection.

The obscuring effects of anonymity not only allowed volunteers to connect to callers past social boundaries, but also accustomed volunteers to the uncertainty of calls and callers’ lives. While anonymity sometimes generalized care, practicing anonymity forced volunteers to embrace differences and limitations within uncertainty to better focus on callers’ perspectives over their own reactions; these efforts volunteers took to listen specifically to each caller also created individual attention and care. When volunteers prioritized and recognized callers as they were over personal ideas of effectiveness, they were better able to facilitate connections across the line—practicing shifting past strangers to belonging.

These care practices that volunteers used to connect with callers helped bridge gaps between strangers in Hong Kong’s urban landscape and political uncertainties. Contrasting the city’s mainstream mental healthcare, which has a background of relying on institutionalization and professional authority, AmaryllisLine’s practice of non-interventive support offered alternative options for care. Given the lack of individual care possible in Hong Kong’s overwhelmed mental health system, volunteers’ practice of listening strived to help callers feel deserving of time and individual attention in a landscape of standardized care. Volunteers’ strategies to listen past prejudice to callers’ personal experiences opened lines for honest conversation and trust building between the growing distances and fractures of an increasingly prevalent stranger society.

We recall Charlie’s words about public apprehensions toward engaging with mental health and his wish for people to gather the courage to step out and help. While it is difficult to find the courage to ask for or give support, hotline callers and volunteers learned not to be daunted and to find curiosity within the unknown. Once made, we can carry the experiences of these connections toward our ‘stepping out’ when we leave the line.

## Footnotes

1 All names are pseudonyms.

2 All interviews were primarily in English.

3 Castle Peak Hospital, established in 1961, was the first psychiatric hospital in Hong Kong. In 1984, Castle Peak established a 24-hour hotline to give advice and crisis intervention, but it mainly became used for education on public mental health (Lo, 2003).

4 AmaryllisLine maintained services during both COVID-19 pandemic and SARS epidemic.

5 Some volunteers joined AmaryllisLine to build resume experience or try out counseling.

6 Many volunteers were surprised by training and unlearned preconceived caregiving notions, speaking to disconnects between public expectations and realities of hotline services.

7 Florence chatted with these callers in Cantonese. Some volunteers expressed naturally becoming more chatty in Cantonese.

8 Some other factors that may contribute to AmaryllisLine’s wide access may include the untethered nature of anonymity, the accessibility of a phone line, and the complementary nature of AmaryllisLine’s service.

9 Volunteers could not disclose shifts to callers.

10 Volunteers had Leaders-on-Shift that they could report to for advice, discussion, and supervision on navigating caller situations as well as personal dilemmas.
